# Supplementary material for: Development and Validation of a Good Manufacturing Process for IL-4-Driven Expansion of Chimeric Cytokine Receptor-Expressing CAR T-Cells
Source: Cells. 2021 Jul 15;10(7):1797. doi: 10.3390/cells10071797 (PMC8307141; doi:10.3390/cells10071797)
Supplement: Supplementary file 1 [file cells-10-01797-s001.zip › cells-1234004-supplementary.pdf]

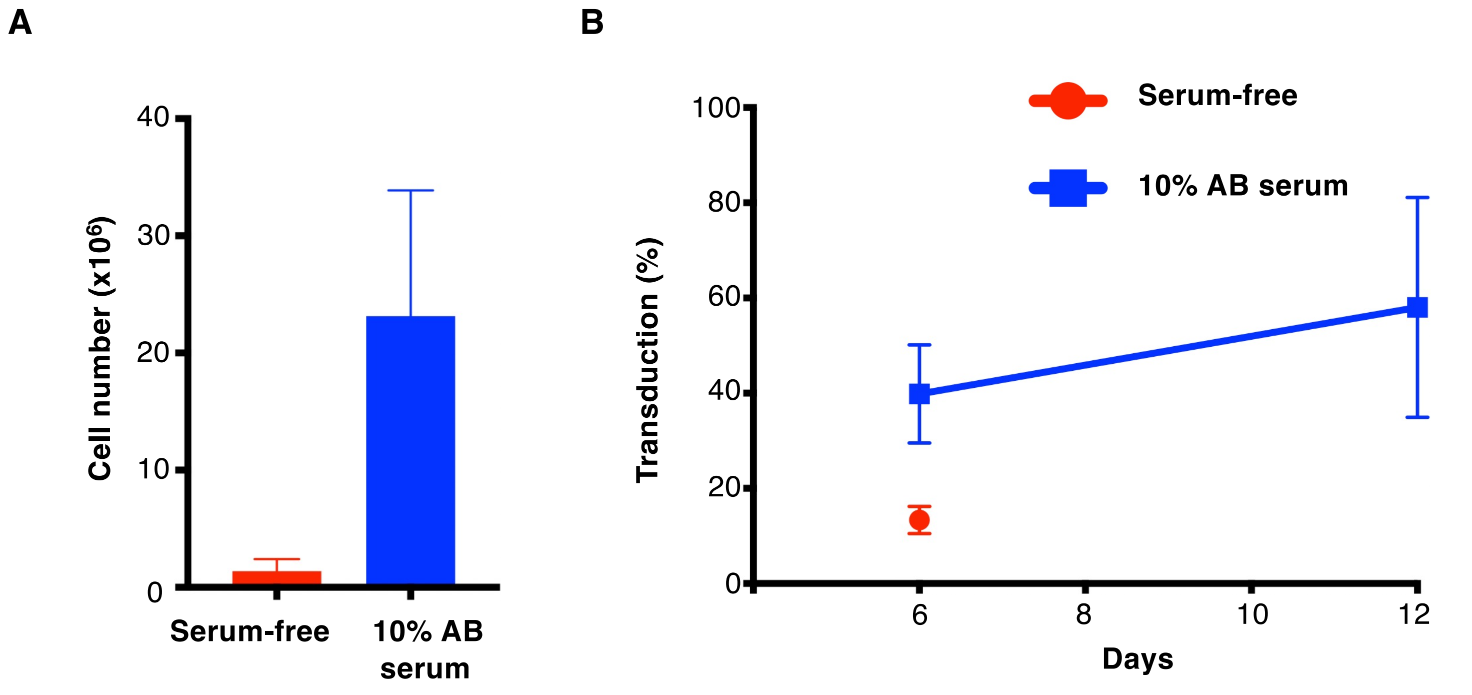

Supplementary Figure S1. Human serum is required for optimum expansion of T4$^+$ T-cells.

Human T-cells (1 million per condition) were transduced with the T4 retroviral vector and then expanded using IL-4-containing X-VIVO^{TM} 15 serum free culture medium or the same medium supplemented with 10% human AB serum. (**A**) The total cell count was measured for both culture conditions on day 11 post transduction. Data shown are the mean ± SD from 3-6 replicates. (**B**) The percentage of CAR$^+$ cells was measured by flow cytometry on days 6 and 12 (latter not possible in the serum-free cultures). Data shown are the mean ± SD from 8 replicates.

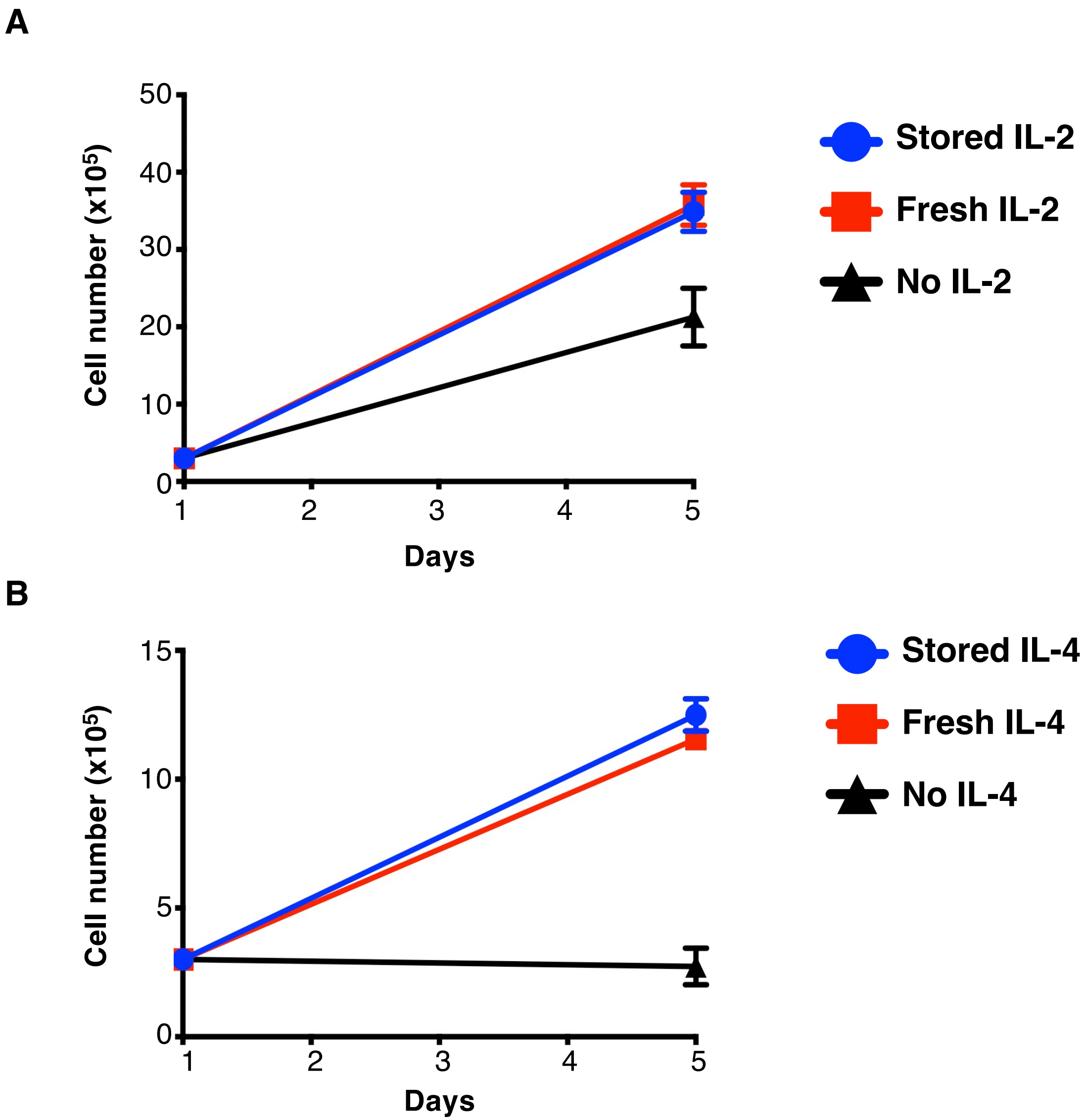

Supplementary Figure S2. Stability testing of cytokine-supplemented complete medium.

(**A**) Complete medium containing IL-2 (Aldesleukin) was prepared fresh or was stored for 4 days at 4ºC. T-cells were activated using CD3 + CD28 beads for 72 hours, mimicking the scenario that applies prior to retroviral transduction. Cultures were divided into 3 aliquots, which were treated with IL-2 (100U/ml), either freshly made or stored at 4°C for 4 days. The third aliquot received no added cytokine. Cell number was evaluated after 4 days (mean ± SD of triplicate cultures). (**B**) Complete medium containing GMP grade IL-4 was newly prepared or was stored for 14 days at 4ºC. T-cells were activated using CD3 + CD28 beads for 72 hours, transduced with SFG T4 and expanded thereafter using IL-4. Cultures were then washed free of exogenous cytokine and divided into 3 aliquots. These were treated with IL-4 (30ng/ml), either freshly made or stored for 14 days at 4ºC. The third aliquot received no added cytokine. Cell number was evaluated after 4 days (mean ± SD n=3 cultures).

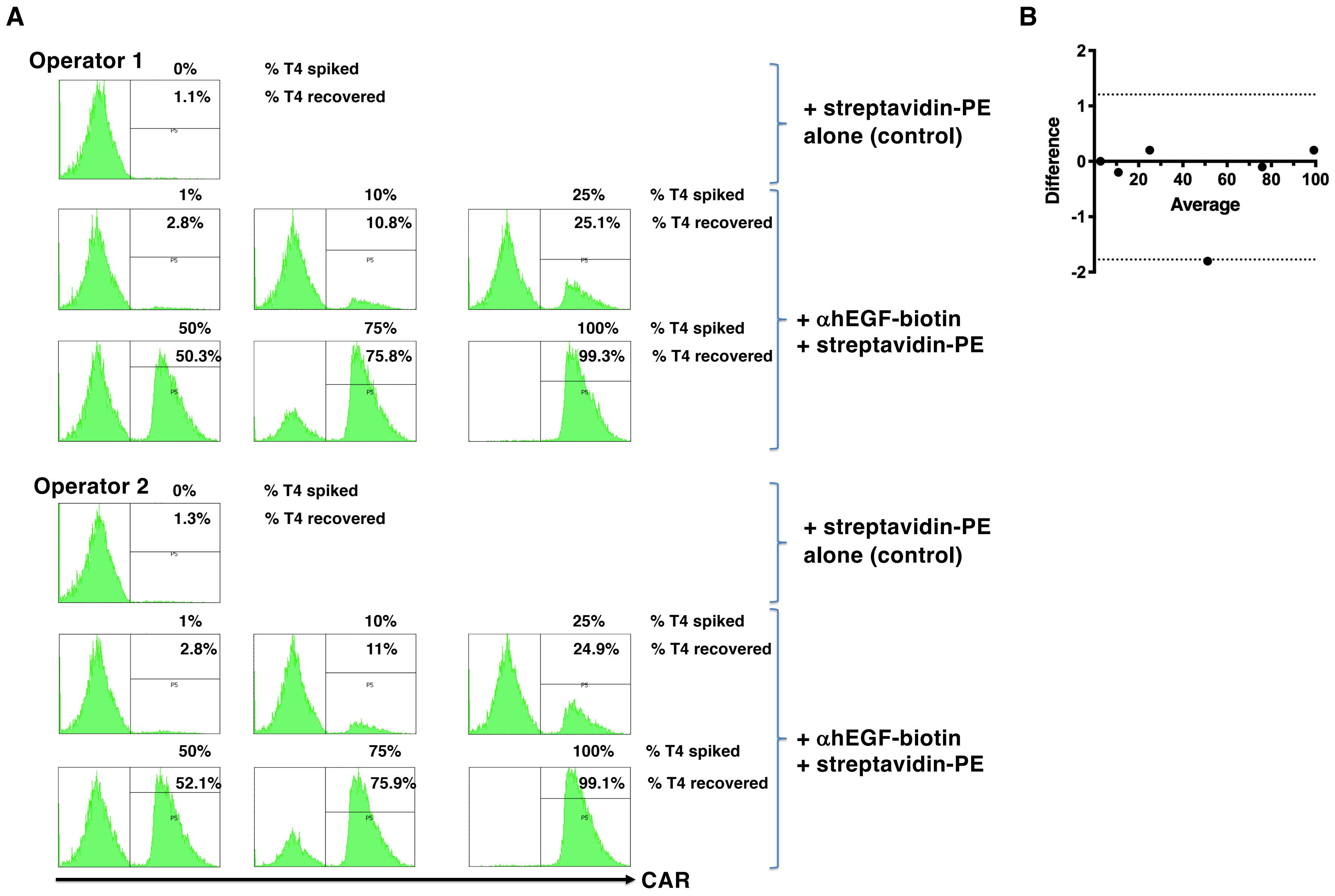

Supplementary Figure S3. Validation of the flow cytometric assay used to detect T4 transgene expression.

(**A**) T4⁺ T-cells were labelled with biotinylated anti-human EGF + streptavidin-PE and were flow sorted to generate a 100% transduced population. These cells were added at known quantities into untransduced autologous T-cells and were then analysed by two independent investigators by flow cytometry in order to detect and quantify the transduced population. (**B**) Bland-Altman plot of differences between both analyses. Note the close agreement between data obtained by both investigators, which match with the predicted proportion of gene-modified cells present. Dotted lines indicate 95% limits of agreement. Data are from a single experiment performed by two operators.

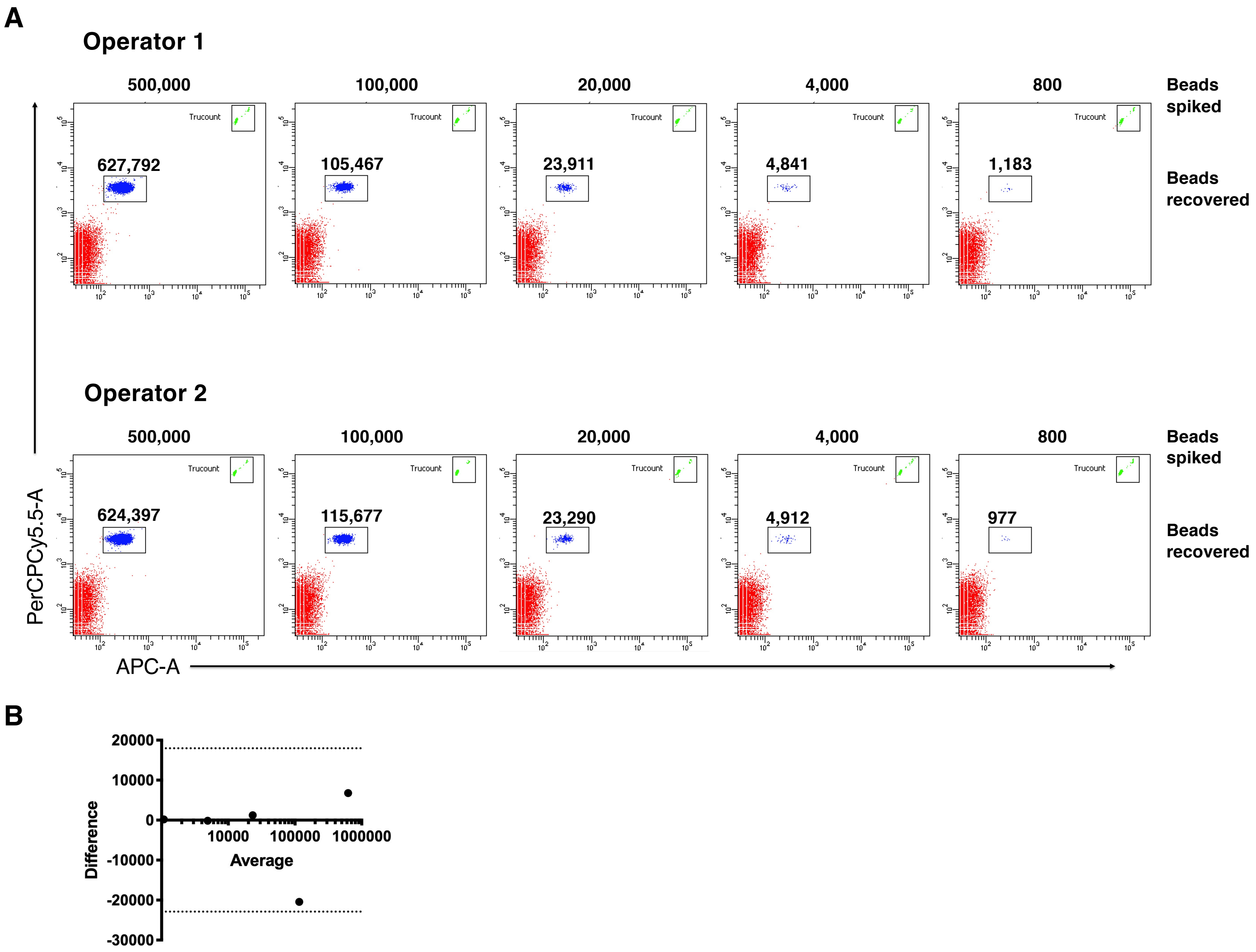

Supplementary Figure S4. Validation of the flow cytometric assay to enumerate residual CD3/CD28 CTS™ Dynabeads®.

(**A**) The indicated known quantity of CD3/ CD28 paramagnetic beads was spiked into a sample of T4 immunotherapy in duplicate. Samples were processed by two independent investigators and absolute number of residual beads detected by flow cytometry. (**B**) Bland-Altman plot of differences between both analyses. Note the close agreement between data obtained by both investigators, which match with the predicted number of beads present. Dotted lines indicate 95% limits of agreement. Data are from a single experiment performed by two operators.

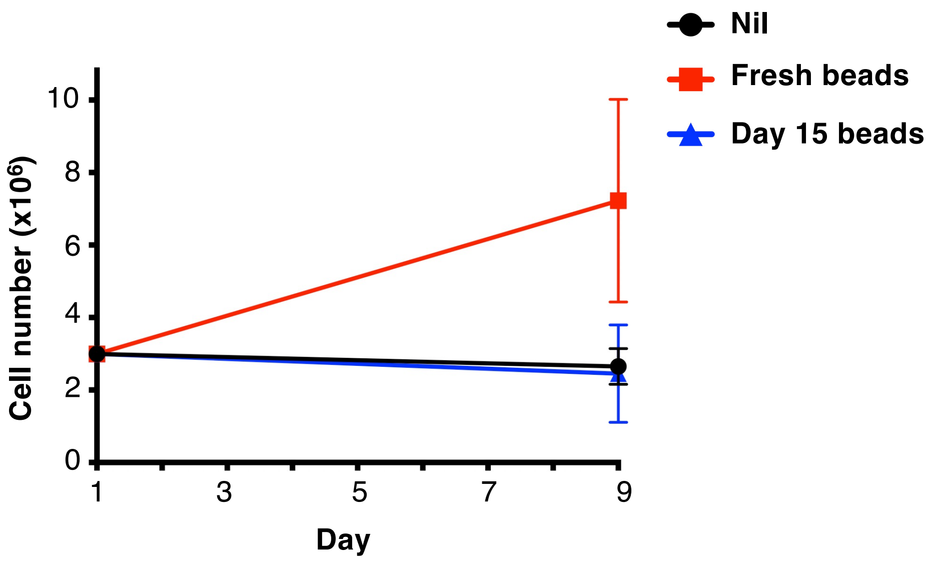

Supplementary Figure S5. Residual CD3/CD28 CTS™ Dynabeads® lack biological activity after manufacture is complete.

Activity of CD3/CD28 paramagnetic beads in the final product (day 15 beads) was compared to that of fresh CD3/CD28 activation beads by analysing their ability to promote T-cell proliferation co-culture with newly isolated PBMCs (mean ± SD , n=2 independent experiments). Cultures were supplemented with complete medium and IL-2 (100U/mL) every 2-3 days.
